# Engineering a smart intrauterine device based on pH‐controlled copper release

**DOI:** 10.1002/btm2.70066

**Published:** 2025-09-06

**Authors:** Greta Bertola, Florinda Coro, Arti Ahluwalia, Carmelo De Maria, Ludovica Cacopardo

**Affiliations:** ^1^ Department of Information Engineering University of Pisa Pisa Italy; ^2^ Research Centre “E. Piaggio” University of Pisa Pisa Italy; ^3^ UBORA Association Pisa Italy

**Keywords:** controlled release, equitable healthcare, intrauterine device, pH sensitive hydrogels, smart medical device

## Abstract

Contraceptive methods based on intrauterine devices (IUD) typically result in women being constantly exposed to either hormones or copper ions. Although it is well known that the pH in vaginal fluids increases from around 3.5 to 7 during intercourse, pH‐responsive materials have yet to be explored for controlling the local release of contraceptive agents. Here, we describe the design of an open‐source smart IUD able to modulate copper ion release on demand thanks to the integration of pH‐sensitive biopolymer‐based hydrogels. Both anionic and cationic hydrogels with different release strategies were investigated. In anionic gels, an increase in pH promotes an increase in the diffusion coefficient; while in cationic gels, an alkaline environment results in shrinking, exposing part of the copper wire. Computational simulations were used to verify that gel thickness was appropriate for minimal copper ion leaching at low pH and effective dose release at higher pH. A thin gel coating was integrated into a commercial IUD using a custom 3D printed mold. Copper ion release was investigated at different time points in acid and basic solutions. The results show that both anionic and cationic gels can be used to engineer smart and safer IUDs.

**Translational Impact Statement**

This proof‐of‐concept study introduces a smart, open‐source intrauterine device (IUD) that uses pH‐sensitive hydrogels to control copper ion release based on physiological pH changes occurring during intercourse. The pH‐responsive polymer gels enhance contraceptive effectiveness while reducing continuous copper exposure. Computational modeling and experimental tests were used to validate the proposed controlled release approach. The open‐source design paves the way for safer and more equitable contraceptive devices which will have a huge impact on women's health worldwide.

## 1 INTRODUCTION

Target 5.6 of the United Nations' Sustainable Development Goals Agenda 2030 aims at ensuring universal access to sexual and reproductive health and reproductive rights.^1^ Contraception is largely targeted at females, and it typically results in a constant exposure to contraceptive substances, with possible side effects on women's health.^2^ Indeed, around 874 million worldwide use modern contraceptive methods, and 164 million have an unmet need for contraception.^3^, ^4^

Intrauterine devices (IUDs) are a widely used method of contraception. They are T‐shaped devices, which are physically inserted into the uterus to prevent pregnancy. IUDs currently on the market are based on the release of copper ions as a spermicidal agent, or of progestin, a hormone that causes thickening of the mucous membrane of the cervix.^3^ In both cases, the presence of a foreign body elicits an inflammatory response which contributes to contraceptive action.^4^ Indeed, copper toxicity and the inflammatory response are responsible for changes in the lining of the uterus preventing oocyte attachment to the endometrium during fertilization.^5^ The copper IUD is also used as an emergency contraceptive because it can prevent up to 99% of pregnancies if inserted within 5 days of unprotected intercourse.^6^, ^7^, ^8^ Recent improvements in IUDs have mainly focused on eliminating problems related to pelvic pain and risk of uterine perforation related to the pressure applied by the device on the uterine wall.^9^, ^10^ Some examples are already available in the market and include copper cylinders piled up in a suture wire sewn to the upper wall of the uterus (GyneFix®, Contrel Europe, Belgium) or copper beads combined with a Nitinol ball‐like structure, which does not require anchoring to the uterus (IUD Ballerine®, OCON Medical Ltd., US).^11^

However, immediately after insertion into the uterus, the high copper corrosion rate can result in a “burst release” of ions (up to 296 μg/day for a 200 mm^2^ Cu‐IUD), causing side effects such as bleeding and pain. After 1–2 months, steady state values in the range of 26–74 μg/day of Cu++ are typical for commercial IUDs.^12^ In fact, in IUD users, mean blood Cu levels were significantly higher (216 μg/dL) than normal (80–160 μg/dL).^12^ Low‐dose copper IUDs have been thus developed, integrating copper elements in strategic locations close to the internal cervix.^13^ Despite a lower copper load than traditional commercial IUDs, exposure to the ions is still constant over time. This induces tissue inflammation and both somatic and psychological symptoms including anxiety, depression, panic attacks, fatigue, heart palpitations, weight gain, hair loss, skin problems, headaches, and insomnia.^14^, ^15^, ^16^ To address these issues, we propose an IUD engineered to release copper only during intercourse.

Normal vaginal pH is slightly acidic (pH 3.5–4.0) but increases up to pH 7.0 within seconds during intercourse. The alkaline vaginal environment is maintained at pH 7 for up to 48 h to ensure sperm survival.^6^ Considering this physiological change in the vaginal environment, pH‐sensitive polymers have emerged as a solution for local “on‐demand” contraception. As an example, ci‐polyvinylic acid and boric acid (Ci‐PVA‐BA) gels were used to create a physical barrier to sperm, switching from a weakly cross‐linked fluidic material to a densely cross‐linked network during intercourse.^15^ Mucoadhesive gels have also been used for improving the local treatment of vaginal infections, such as bacterial vaginosis and candidiasis^17^ or for the systemic release of anti‐HIV drugs.^18^ These formulations, which are based on alginate or a blend of poly(lactic‐co‐glycolic acid) (PLGA) and methacrylic acid copolymer, increase the residence time at the site of action with respect to standard delivery systems such as capsules, creams, and ovules.^19^

Despite their potential, to date, to the best of our knowledge, pH‐sensitive materials have not been exploited to modulate the release of contraceptive copper ions in IUDs such that women are exposed to the contraceptive agent only when necessary. To address this gap, we conducted a proof‐of‐concept study on the application of pH‐responsive gels for the modulation of copper ion release by coating IUDs with either polyacid (anionic) or polybasic (cationic) gels. We considered two pH‐responsive gels widely employed for controlled‐release formulations in biomedical applications, polyacrylamide (PAAm) and chitosan.^20^ The radical copolymerization of acrylamide monomers (AAm) and N,N‐methylenebis (bis‐acrylamide) forms negatively charged polyacid gels with high stability in aqueous environments. The molar ratio of the monomer to the crosslinker ranges from 10 to 10^4^ and the volume fraction of water ranges from 70% to 90%.^21^, ^22^, ^23^ Chitosan is a linear polysaccharide, composed of randomly distributed D‐glucosamine and N‐acetyl‐D‐glucosamine units. This material is obtained by partial deacetylation of naturally available insoluble chitin obtained from exoskeletons of crustaceans, fungi, and insects. It can be used for the fabrication of nontoxic, stable, and biodegradable polybasic, that is, positively charged, gels.^24^, ^25^, ^26^ Starting from these two well‐known polymers, we investigated different coating designs to minimize ion release at physiological acid pH by hindering ion diffusion, while ensuring the release of efficacious spermicide ion concentrations (10^−6^ M^11^) as soon as the pH increases.

As female contraception, particularly that which provides long‐term protection against unwanted pregnancies, is widely used in low‐ and middle‐income countries, we pursued an open‐source approach for the design to promote equitable healthcare.^27^ To this end, the UBORA platform was used to guide the study design, beginning with an assessment of the current needs for contraception^28^ geared toward seeking solutions which better preserve woman's health and concluding with a cost–benefit analysis.

## 2 RESULTS AND DISCUSSION

### 2.1 Device classification using UBORA

We used the UBORA pipeline to identify the risk class of the device. According to rule 15 of Annex VIII of the European Parliament and European Council Regulation (EU) 745/2017 (MDR 745/2017) and the classification tool of the UBORA platform, the device was classified as a Class III medical device. Risk traceability matrix and compliance with safety and performance requirements of Annex I of MDR 745/2017 are reported in the Supporting Information.

### 2.2 Preliminary market evaluation and cost–benefit analysis

IUD market size^29^ is estimated to reach 9.29 USD billion by 2034 with a compounded annual growth rate (CAGR) of 3.67%. However, although current copper IUDs are highly effective, they are often associated with user discontinuation due to continuous copper release and side effects such as increased bleeding and cramping. The proposed innovative pH‐responsive gel‐coated copper IUD can further support this market, improving targeted contraceptive action while minimizing side effects. Indeed, a device that selectively releases copper only when necessary (during the intercourse) could improve tolerability and long‐term use. Should the smart release mechanism be proven to reduce adverse effects, the added value may justify a slightly higher manufacturing cost. Additionally, fewer follow‐up appointments and removals may result in overall cost savings for healthcare systems and hence a favorable cost–benefit profile.

### 2.3 Stakeholder analysis

The target population for this device includes all women of reproductive age, considering that the proposed improvement of a commercial product, being safer and less toxic, would be used more widely than now. However, the development and adoption of this device must consider a range of stakeholders beyond the end user. Healthcare providers (e.g., gynecologists and family planning specialists) play a critical role in recommending and managing contraceptive devices. Their acceptance depends on ease of insertion, reliability, and reduced complication rates. Employees and insurance providers may be more likely to support coverage for a device that reduces adverse events and follow‐up care. Moreover, public health agencies may be interested in a novel IUD that improves compliance and expands contraceptive options, especially in low‐resource settings.

### 2.4 pH‐responsive behavior and stability of PAAM and chitosan

**FIGURE 1:**
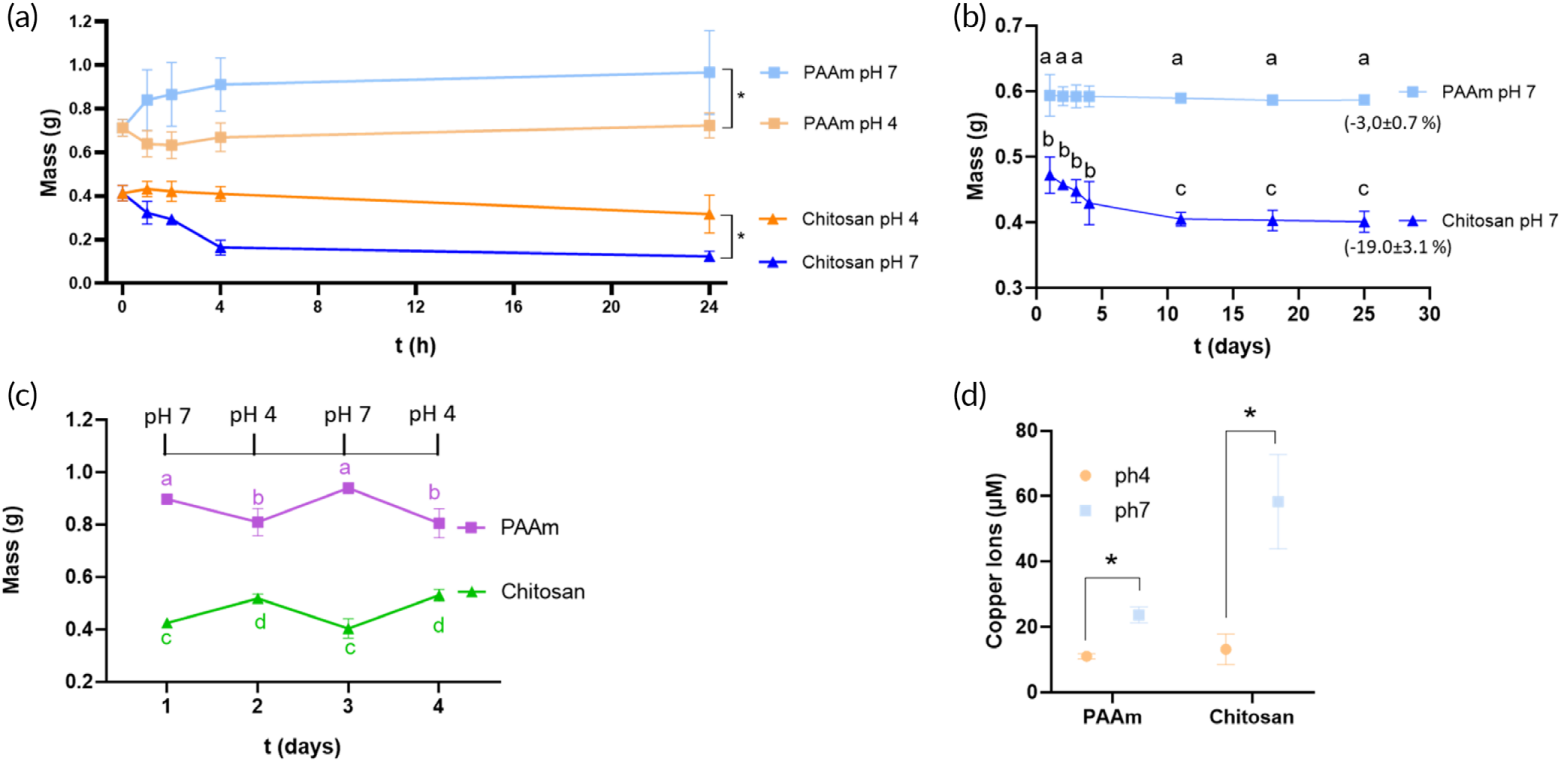
(a) Average mass of the PAAm and chitosan samples at pH 4 and pH 7 at different time points. (b) PAAm and chitosan degradation (mass loss) over 25 days. Values in brackets outline mass percentage variation at day 25 with respect to day 1. (c) Cyclic pH variations. Different letters and * indicate statistically significant differences (*ƿ* < 0.05). (d) Experimental Cu^2+^ ion concentration released from IUDs wrapped by a 2 mm PAAm and chitosan layer after 8 h in buffer solutions at pH 4 or 7.

The swelling ratio was defined as the mass of the gel at pH 7 with respect to its mass at pH 4, expressed as a percentage. As expected, the mass of the PAAm gel increased with increasing pH, while the mass of the chitosan gel decreased. As shown in Figure 1a, PAAm samples started swelling immediately on contact with the buffer solution at pH 7 and reached a plateau after 4 h. However, they remained almost constant in weight at pH 4. Chitosan gels underwent rapid shrinking over 4 h in pH 7 buffer, after which the rate of water loss decreased. The samples also underwent a gradual decrease in mass over 24 h at pH 4. The swelling ratios of PAAm and chitosan at 24 h were 34% ± 26% and −61% ± 8%, respectively. Long‐term degradation tests (Figure 1b) showed that chitosan mass decreased, reaching a plateau around day 10, while PAAm mass was constant over 25 days. Cyclic tests also showed the reversibility of the process (Figure 1c).

### 2.5 Copper release efficiency

**FIGURE 2:**
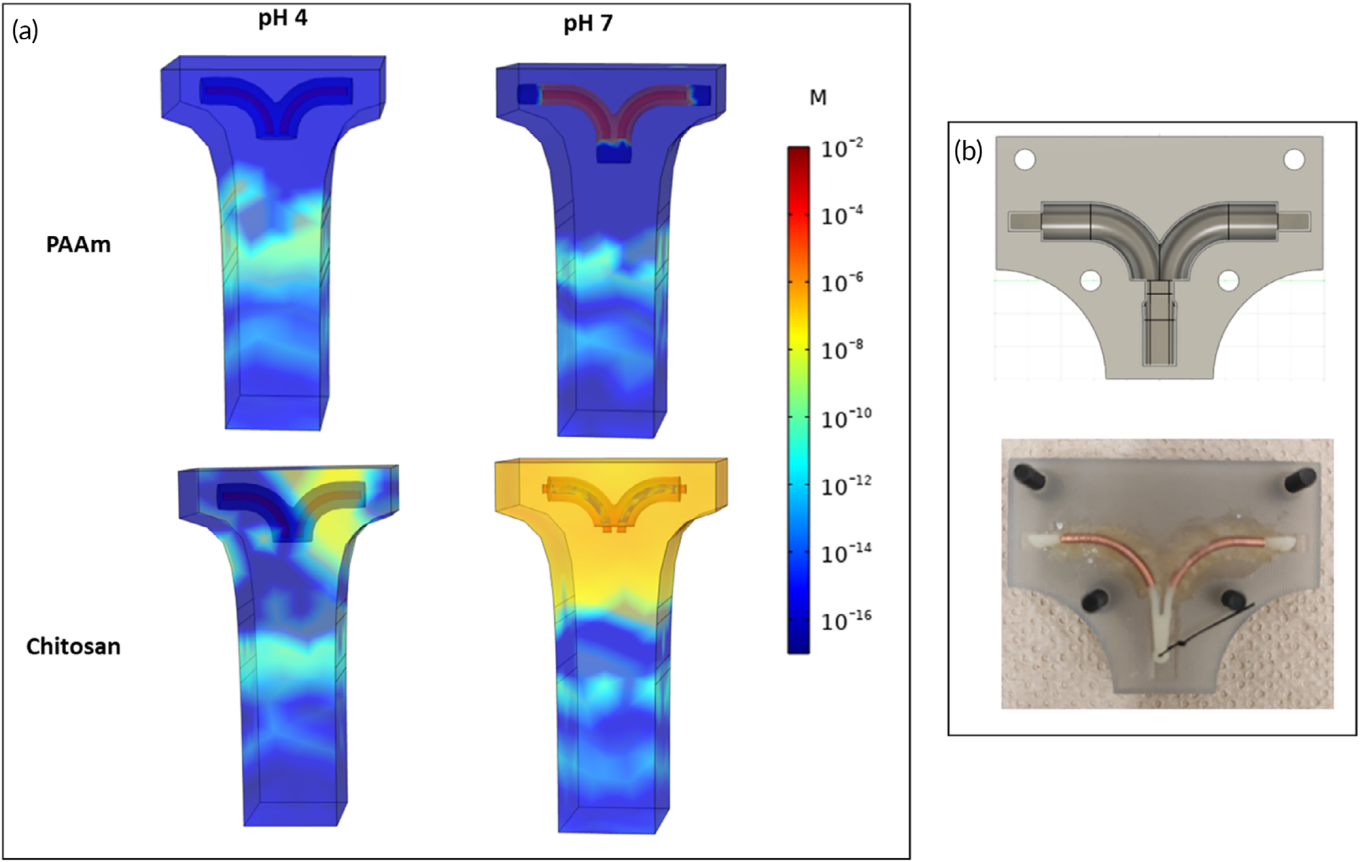
(a) Computational simulations showing copper ion concentration (M) after 8 h at pH 4 (left) and pH 7 (right) for a 2 mm layer of PAAm (first row) and chitosan (second row). (b) Blown‐up geometry of the IUD device.

Computational predictions demonstrated that a layer of gel with thickness between 1 and 2 mm limited copper release at pH 4 and reached the necessary dose for spermicide action (10^−6^ M) at pH 7 (Figure S1). The validation experiments were thus conducted on 2 mm thick gels because it allows for the fabrication of uniform gels, as the polymer can be better distributed in the mold during casting (Figure 2b).

As shown in Figure 2a, at pH 4 and for both polymers, ions were confined at the copper surface and do not reach high concentrations in the solution because of the polymer barrier. At pH 7, the ions diffuse through the PAAm swollen network, reaching higher concentrations. In the case of chitosan, ion release at pH 7 is ensured by gel shrinking and exposure of the IUD copper element. The computed average concentration in the fluid at pH 7 was equal to 27 × 10^−6^ M in the case of PAAm and 35 × 10^−6^ M in the case of chitosan. The experimental ion concentration at pH 7 is coherent with predictions from mathematical simulations. The slightly higher experimental Cu++ ion concentrations observed for polybasic gels are likely associated with chitosan degradation, which was not considered in the computational setup.

Although both gels were effective in modulating copper ion release according to pH variations, PAAm presented a higher stability in wet environments, which may foster its further development in clinical applications. However, the toxicity of the acrylamide monomer makes chitosan a completely natural, stable, and low‐risk solution worthy of consideration. For both solutions, additional development should consider the optimization of a robust supply chain, including suitable device packaging for maintaining gel hydration and sterility until implantation.

Although the 8 h simulation considers the typical time‐window for which vaginal pH is altered after intercourse,^30^ future studies should also address the effective ion concentration and spermicide efficacy in a longer time‐window. The tests performed using buffered saline solutions were conducted to investigate the operation of the materials in this proof‐of‐concept study. Modeling more realistic scenarios using simulated uterine and seminal fluids (SUF and SSF, respectively) will be essential for more in‐depth investigations of the device's performance. These fluids have complex chemical compositions which could modulate or interfere with copper ion release. For instance, SUF is based on a combination of different salts, bovine serum albumin, lactic acid, acetic acid, and glucose, while SSF contains different salts, acid phosphatase, ascorbic acid, prostaglandins, l‐carnitine, and alpha‐glucosidase. Commercial formulations are available but can be costly for low‐resource settings ($275 and $975 for 500 mL of SUF and SSF, respectively). However, they can also be prepared in the lab using published protocols.^31^, ^32^, ^33^ Finally, further validation of the system should also consider more realistic conditions such as fluid flow and physiological temperature.

## 3 MATERIALS AND METHODS

### 3.1 Conceptual design

The design was supported by the UBORA platform,^34^, ^35^ a tool for the collaborative design of certifiable medical devices compliant with MDR 745/2017 on medical devices.^36^ UBORA provides a structured design framework, divided into Work Packages (WPs). In particular, WP‐1 focuses on fundamental aspects prior to effective design, such as clinical need, analysis of existing solutions, intended user, and product requirements.

The clinical need of this study was: “*improving the health status of women using the copper IUD, limiting exposure to toxic copper ions to only those cases where it is necessary* (*during intercourse*)”. As already stated in the introduction, once the commercial copper‐releasing IUD is implanted, the woman is constantly subject to the release of copper ions that causes constant uterine inflammation and inhibits the attachment of the fetus to the wall. Here we propose enhancing a commercial l IUD by covering the copper parts with a pH‐sensitive gel that allows ion release only when the pH changes. In addition to the technical specifications of the device (minimum concentration for contraception, ease of insertion, sterility, etc.), it was appropriate to assess the technical characteristics of the gel used for modulating the release, which must be stable at 37°C for a very long period (IUDs are implanted for about 3–5 years) and must guarantee a controlled pH‐dependent swelling that inhibits/favors the release of copper ions. Moreover, the target copper concentration for effective spermicide action during the intercourse should be in the range of 10^−6^ M.^11^ The pH‐responsive gel should exhibit rapid and reversible structural changes in response to physiological pH variations, contracting or swelling to expose or enhance the diffusion of copper ions during intercourse and returning to a stable state otherwise. Additional specifications to be tested in the pre‐industrialization phase are gel long‐term structural stability and adhesion to the copper surface to remain functional under uterine mechanical stress and fluid exposure for a lifespan of 3–5 years.^37^

Alongside these aspects, in WP‐1, the UBORA platform provided support in classifying the device according to Annex VIII of EU MDR and identifying international standards useful to meet the general safety and performance requirements of Annex I of the same regulation.

### 3.2 Principle of operation

**FIGURE 3:**
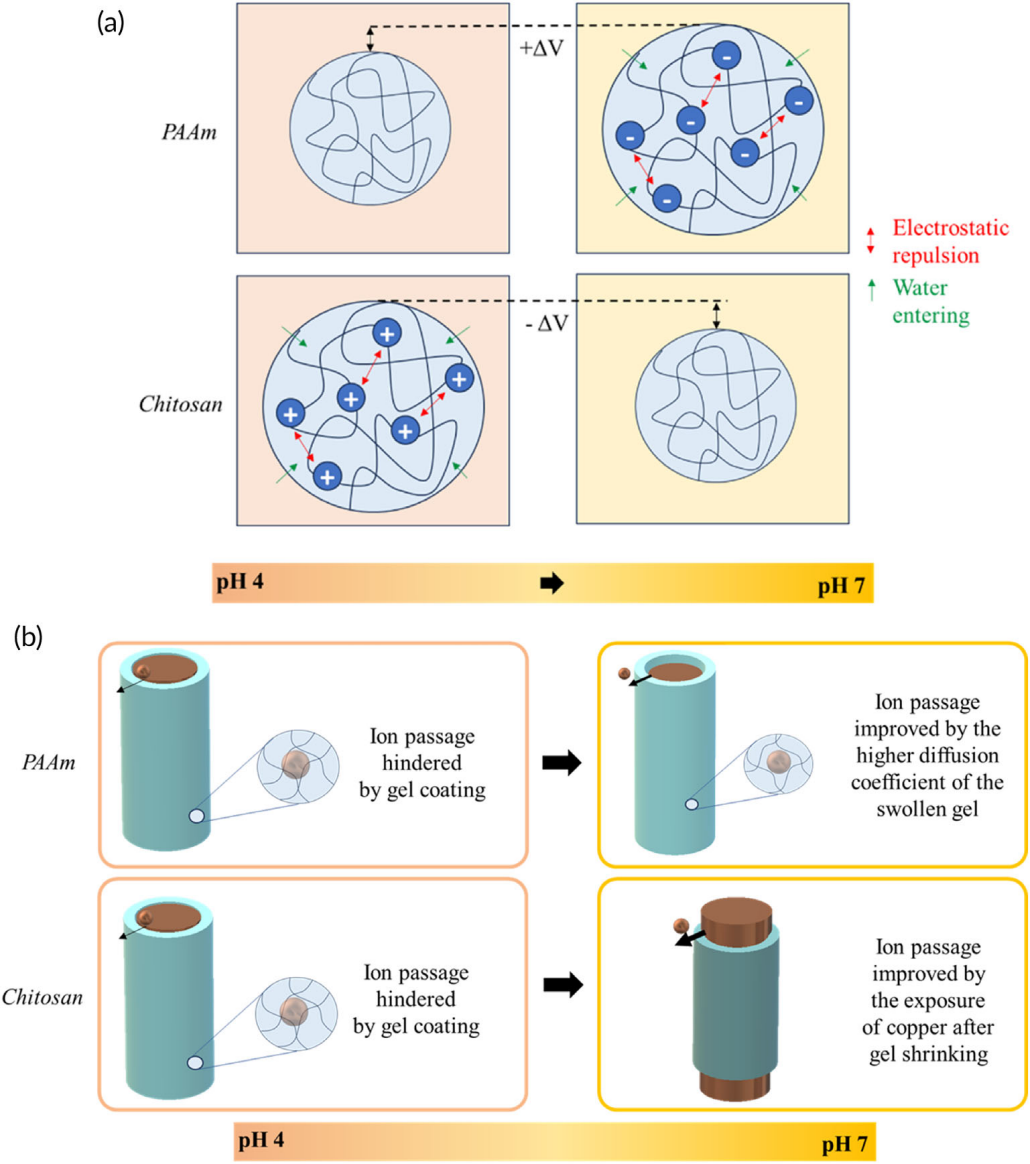
Schematic representation of (a) PAAm and chitosan swelling and shrinking with increasing pH; (b) the smart IUD coated with PAAm and chitosan gels.

The principle of operation of the proposed IUD is based on changes in gel ionization in response to environmental pH. Polyacid polymers, such as PAAm, swell at high pH because of the ionization of carboxylic pendant groups. The repulsion between negative ionized groups is accompanied by an increase in network mesh size and water absorption (Figure 3a). Alternatively, polybasic polymers, such as chitosan, present positively charged aminic groups at low pH and deionize in alkaline environments. The process is thus associated with water expulsion, a reduction in mesh size, and gel shrinking (Figure 1a). Swelling is generally accompanied by the uptake of water‐soluble molecules, while shrinking is associated with the release of hydrophilic constituents.^38^, ^39^, ^40^, ^41^, ^42^, ^43^, ^44^

At low pH, the polymeric coating protects the copper element from the acid environment limiting Cu^2+^ diffusion by steric hindrance. In addition, at pH 4, chitosan is a good sorbent of copper ions, thereby reducing their diffusion.^45^, ^46^

An increase in pH results in swelling of the PAAm gel; the mesh size increases, and copper ions diffuse more easily through the gel. On the other hand, chitosan will shrink when the pH increases, exposing part of the copper element. Copper ions are thus released into the environment. Figure 3b illustrates this concept.

### 3.3 Materials

NO‐Gravid® IUDs were kindly provided by I.R.MED. S.r.l., Italy. Acrylamide, ammonium persulfate, TEMED, chitosan, glutaraldehyde (GTA), sodium phosphate dibasic, citric acid, phosphate buffered saline, and acetic acid were purchased from Merk, Italy. Bis‐acrylamide was purchased from Biorad, USA. The Copper (Cu) Colorimetric Assay Kit (E‐BC‐K300‐M) was purchased from Elabscience, USA.

### 3.4 Gel swelling and degradation tests

A 15.6% w/v Acrylamide, 0.02% w/v Bis‐acrylamide (Bis), 0.086% w/v Ammonium persulfate, and 0.056% v/v TEMED solution was prepared and degassed for 2 min. Then, the solution was poured into custom cylindrical molds (diameter = 13 mm, height = 4 mm) and incubated at room temperature for 90 min. A 1% w/v Chitosan solution in 2% w/v Acetic Acid and 0.5% v/v GTA were prepared and poured into the same molds, which were placed in the freezer (−20°C) overnight.

After gelation, samples were moved to 12‐well plates and submerged in 2 mL of pH 4 and 7 buffer solutions prepared using sodium phosphate dibasic 0.2 M and citric acid 0.1 M (respectively 39%–61% v/v and 82%–18% v/v). Gel height and diameter variations were measured with a vernier caliper and weighed at different time points (1, 2, 4 and 24 h).

Cyclic swelling tests were also performed over 4 days to assess the reversibility and repeatability of the swelling/deswelling process with pH. Using the same experimental setup, the gels were alternatively placed in solutions at different pH, switching among the two conditions and measuring the relative mass variations daily. Finally, samples were placed in pH 4 buffer solution and weighed at different time points over 25 days to test the stability of the gels in a physiological vaginal‐like pH environment.

### 3.5 Computational studies

**FIGURE 4:**
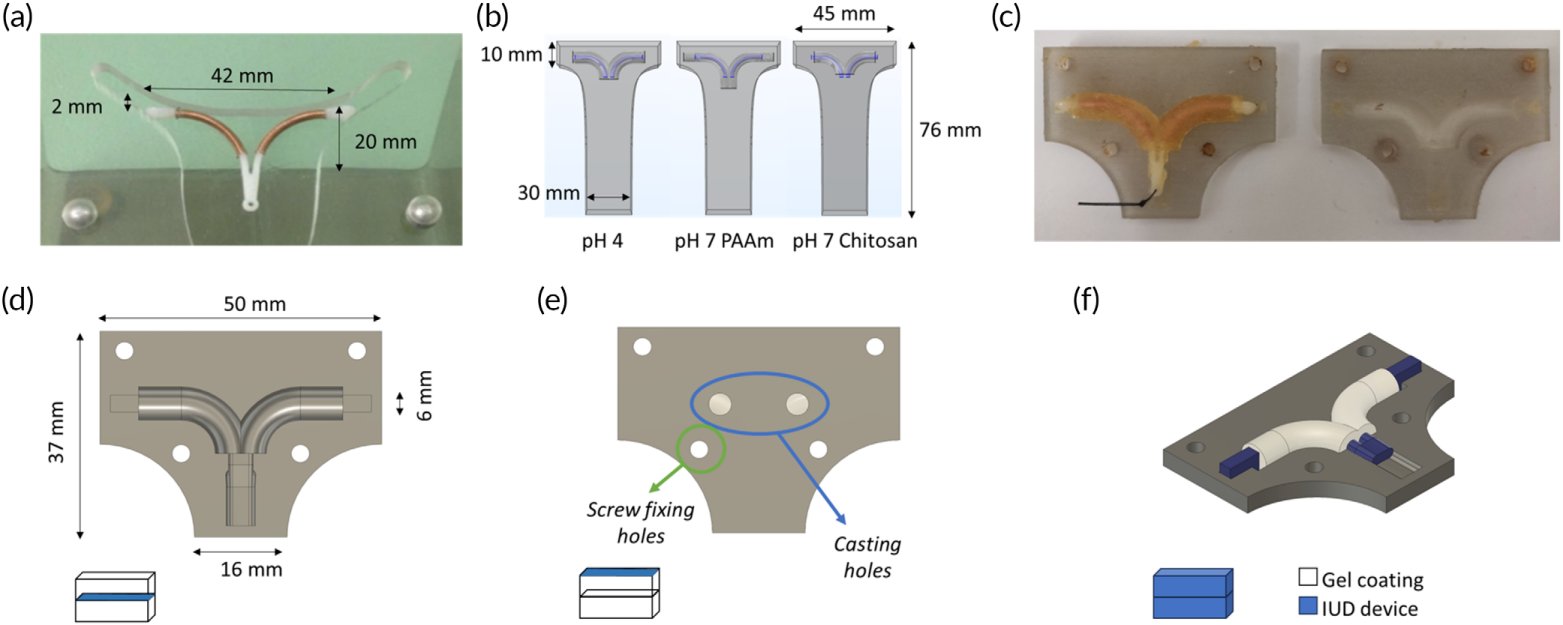
Schematic geometry and average dimensions of uterus for the FEM model. (a) NO‐Gravid® IUD in a phantom uterus; (b) Model geometry for the gels at the different pH; (c) Pictures of the mold with gel‐coated IUD; (d, e) Drawings of the mold; (f) Principle of operation of the mold.

To simulate the process of copper ion release from an IUD, a finite element method (FEM) model was developed in COMSOL Multiphysics® using the Dilute Species Transport Module. The commercial copper IUD NO‐Gravid® was employed as a case study. The geometry was defined using Autodesk Fusion 360® considering the geometry of a typical uterus and the IUD (Figure 4a,b).^47^ A gel layer was designed around the copper elements on the IUD arms, considering a 50 μg/day average ion release.^11^, ^48^ The pH‐dependent Cu^2+^ diffusion coefficients in the two gels were taken from the literature (Table S1). Radial and axial hydrogel variations derived from the experimental swelling tests and calculated as the percentage variation with respect to the dimensions at pH 4 were also considered, as reported in Table 1. The higher axial swelling/shrinking with respect to radial dimensional variation is coherent with other results in the literature which report anisotropic shrinking in cylindrical gels.^49^, ^50^ A “No Flux” boundary condition was selected for the uterus wall (Figure 4b), while “Surface Reaction” was set for the IUD copper surface in which we have an outgoing ion flux of 4.5 × 10^−8^ (mol/m^2^ s). A parametric sweep was thus performed considering gel thicknesses from 1 to 2 mm with a 0.25 mm pitch. These values were selected as the minimum and maximum thickness for gel stability, handling, and encumbrance. Copper concentration was calculated after 8 h as it represents the typical time window for which vaginal pH is altered after intercourse.^30^

**TABLE 1:** axial and radial variation at pH 7 with respect to pH 4.

Material	Axial (%)	Radial (%)
PAAm	+27.6	+14.1
Chitosan	−28.6	−18.1

### 3.6 Fabrication and testing of smart IUDs

TGels were cast into a 3D printed custom mold to obtain a uniform gel coating layer around the copper element of the IUD. The mold consisted of two parts (top and bottom) which were designed on Autodesk Fusion360® and prototyped in Tough resin 1500 using the stereolithographic printer Form 3 (Formlabs, USA). The bottom part (Figure 4d–f) was first prefilled with polymer solution. Then the IUD device was positioned and covered with the top, which was tightened using screws. Finally, casting holes in the top part (Figure 4e) were used to completely cover the copper elements. The gels were prepared following the protocols described in Section 2.1 for PAAm and chitosan (Figure 4c).

After their extraction from the molds, the smart IUDs were submerged in 10 mL of pH 4 or 7 buffer solution for 8 h. Copper ion release was measured using the Copper (Cu) Colorimetric Assay.

All experiments were performed in triplicate and expressed as mean ± standard deviation. Statistical analysis (2‐way ANOVA, followed by Tukey's multiple comparison) was performed using GraphPad Prism software.

## 4 CONCLUSIONS

Given the persistent gender imbalance in contraceptive options, where women bear the primary burden and health risks, this study represents a significant step toward addressing this inequity. By introducing a novel approach that utilizes pH‐sensitive gels to regulate copper ion release, we have demonstrated a promising proof of concept for a smarter IUD that minimizes continuous copper exposure while ensuring effective contraception post‐intercourse. This innovation holds the potential to greatly reduce side effects associated with long‐term copper exposure. Our user‐oriented design workflow, empowered by the UBORA platform, allowed the definition of a smart contraceptive solution that is also sustainable, accessible, and safe, promoting the wide use of contraceptive devices and contributing to an overall improvement in women's health worldwide, particularly in low‐ and middle‐income countries where access to a wide range of contraceptive options is limited.

While our initial findings are encouraging, key challenges remain, particularly in the stabilization of the chitosan gel, device packaging, sterilization, and the development of a robust supply chain. However, with further refinement and development, this technology could usher in a new era of safer, more responsive contraceptives, empowering women worldwide with greater control over their reproductive health.

## CONFLICT OF INTEREST STATEMENT

Arti Ahluwalia and Carmelo De Maria are members of the UBORA Association.

## Supporting information


**Data S1.** Supporting Information.

## Data Availability

The data that support the findings of this study are available from the corresponding author upon reasonable request.
