# Supplementary material for: Engineering a smart intrauterine device based on pH‐controlled copper release
Source: Bioeng Transl Med. 2025 Sep 6;10(6):e70066. doi: 10.1002/btm2.70066 (PMC12617541; doi:10.1002/btm2.70066)
Supplement: Supplementary file 1 — Data S1. Supporting Information. [file BTM2-10-e70066-s001.zip › btm270066-sup-0007-TableS1-S2-FigureS1@Supplementary material_rev 24.06.docx]

**Engineering a smart intrauterine device based on pH-controlled copper release**

Greta Bertola^1^, Florinda Coro^1,2^, Arti Ahluwalia^1,2,3^, Carmelo De Maria^1,2,3,^ °, Ludovica Cacopardo^1,2,°,*^

^1^ Department of Information Engineering, University of Pisa, Italy

^2^ Research Centre “E. Piaggio”, University of Pisa, Italy

^3^ UBORA Association, Italy

° These authors share last authorship

* Corresponding author, [ludovica.cacopardo@unipi.it](mailto:ludovica.cacopardo@unipi.it)

**Supplementary Material**

| Material | Diffusion coefficients [m^2^/s] | |
| --- | --- | --- |
|  | @ pH 4 | @ pH 7 |

| PAAm | 6.3*10^-12^ | 5.8*10^-11^ |
| --- | --- | --- |
| Chitosan | 3.3*10^-12^ | 3.3*10^-13^ |

Table S1: Diffusion Coefficient [1-2]


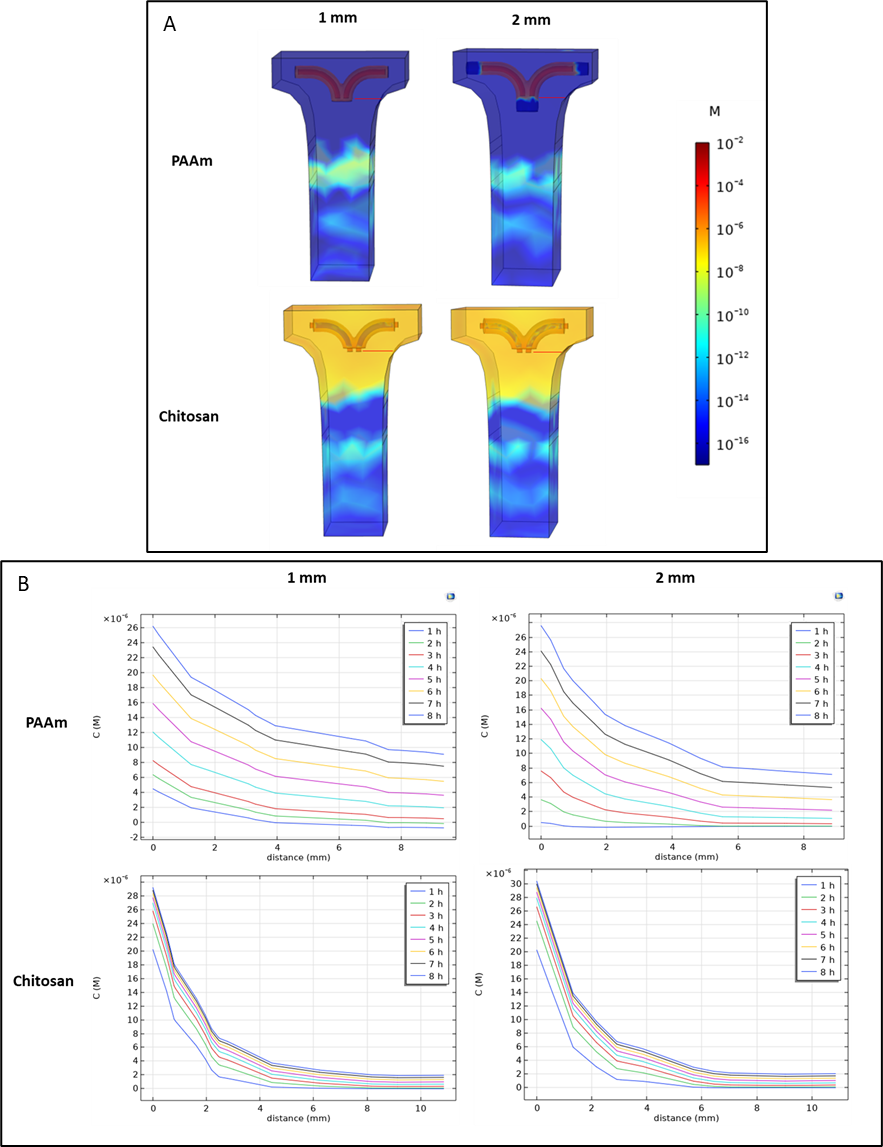


Figure S1: Comparison between different gel thicknesses at pH 7: surface (A) and line plots (B) showing copper ion concentration. Cut lines are indicated in red.

**Analysis of Device Risk Classification and Conformity to the EU MDR Regulation**

IUDs can be classified as class III medical devices in accordance with Rule 15 of Annex VIII of the Medical Device Regulation (MDR) 745/2017 [3].

The regulatory aspects of the device were evaluated using the regulation checklist tool of the UBORA Platform [4], [5], [6]. The following are some of the standards that should be used to ensure compliance with Annex I of MDR 745/2017 regarding safety and performance requirements as required by the CE marking process:

- ISO 14630:2012 NO Active surgical implant;
- ISO 7439:2023 Copper-bearing contraceptive intrauterine devices —Requirements and tests;
- ISO 14971:2019 Medical devices — Application of risk management to medical devices;
- ISO 10993-1:2020 Part 1: Evaluation and testing within a risk management process;
- ISO 10993-3:2014 Part 3: Tests for genotoxicity, carcinogenicity and reproductive toxicity;
- ISO 10993-5:2009, Biological evaluation of medical devices - Part 5: Tests for in vitro cytotoxicity;
- ISO 10993-10:2023, Biological evaluation of medical devices – Part 10: Tests for skin sensitization;
- ISO 10993-11:2018 Part 11: Tests for systemic toxicity;
- ISO 13485:2016 Medical devices — Quality management systems —Requirements for regulatory purposes;
- ISO 11137‐1:2015+A2:2019 Part 1: Requirements for development, validation and routine control of a sterilization process for medical devices;
- ISO 11137-2:2015 Part 2: Establishing the sterilization dose;
- ISO 13408-1:2015 Aseptic processing of healthcare products;
- ISO 15223-1:2021 Medical devices — Symbols to be used with information to be supplied by the manufacturer — Part 1: General requirements;
- ISO/TR 24971:2020 Medical devices — Guidance on the application of ISO 14971;

Risk analysis was performed referring to guidelines of standard ISO 14971:2019. The risks (R) associated with hazardous situations were estimated by multiplying the severity index (S) and the probability of occurrence (O) and risk mitigation (RMC) index (Table S2). Then, a 5x5 risk matrix was used. Considering R≤4 as a threshold for risk acceptability, the identified risks resulted acceptable. Only a small excerpt of possible risks related to the use of the medical device, as an example, are shown in Table S2.

| Hazardous situation/ foreseeable sequence of event | Harm | O | S | RMC | RMCindex | R |
| --- | --- | --- | --- | --- | --- | --- |
| Wrap damage, non-sterile IUD | Microbiological contamination | 1 | 1 | Enclosure condition check | 0.4 | 0.4 |
| Non-sterile IUD after sterilization process | Microbiological contamination | 1 | 1 | Sterility control | 0.4 | 0.4 |

Table S2: List of main risk (R risks; S severity; O probability of occurrence; RMC risk mitigation index).

# Bibliography

[1] Bajpai, S. K., & Dubey, S. (2004). Modulation of dynamic release of vitamin B2 from a model pH‐sensitive terpolymeric hydrogel system. *Polymer international*, *53*(12), 2178-2187.

[2] Chu, K. H. (2002). Removal of copper from aqueous solution by chitosan in prawn shell: adsorption equilibrium and kinetics. *Journal of Hazardous Materials*, *90*(1), 77-95.

[3] The European Parliament and European Council. Regulation (EU) 2017/745 on Medical Devices

[4] De Maria C, Di Pietro L, Diaz Lantada A, et al. The UBORA E-Infrastructure for Open Source Innovation in Medical Technology. In: XV Mediterranean Conference on Medical and Biological Engineering and Computing – MEDICON 2019. 2020:878-882. doi:10.1007/978-3-030-31635-8_106

[5] De Maria Carmelo and Díaz Lantada A and Di Pietro Licia and Ravizza Alice and Arti Ahluwalia. Open-Source Medical Devices: Concept, Trends, and Challenges Toward Equitable Healthcare Technology. In: Ahluwalia Arti and De Maria C and DLA, ed. Engineering Open-Source Medical Devices: A Reliable Approach for Safe, Sustainable and Accessible Healthcare. Springer International Publishing; 2022:1-19. doi:10.1007/978-3-030-79363-0_1

[5] UBORA: Open Biomedical Engineering e-platform for Innovation through Education – On line resources, available at <https://platform.ubora-biomedical.org> [last access June 2025]
