# Supplementary figures and images for: Engineering a smart intrauterine device based on pH‐controlled copper release
Source: Bioeng Transl Med. 2025 Sep 6;10(6):e70066. doi: 10.1002/btm2.70066 (PMC12617541; doi:10.1002/btm2.70066)

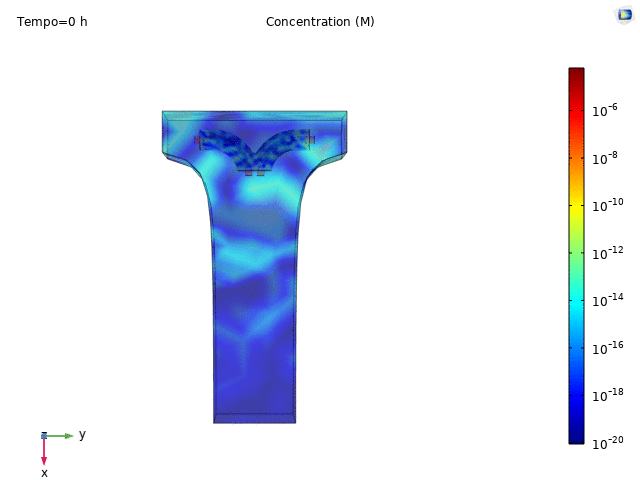

Supplement: Supplementary file 1 — Data S1. Supporting Information. [file BTM2-10-e70066-s001.zip › btm270066-sup-0001-Supinfo1@coating chitosano pH 7.gif]

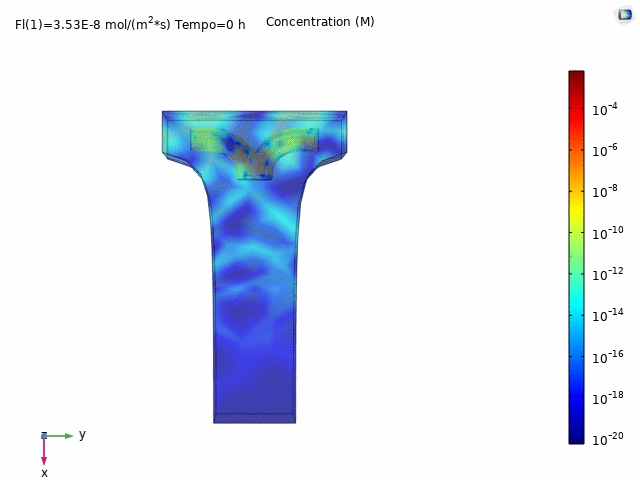

Supplement: Supplementary file 1 — Data S1. Supporting Information. [file BTM2-10-e70066-s001.zip › btm270066-sup-0002-Supinfo2@coating chitosano pH4.gif]

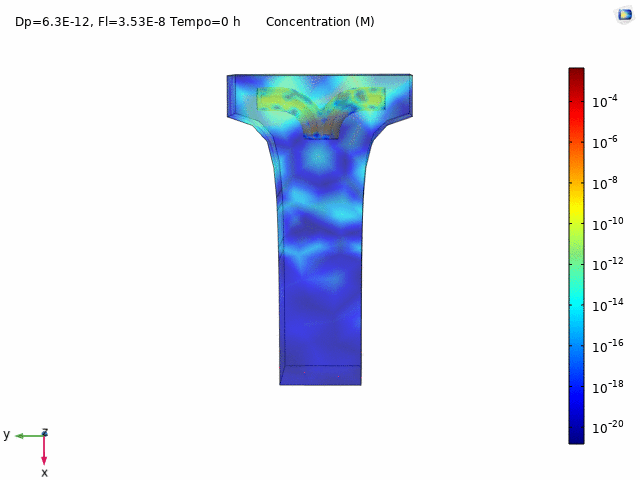

Supplement: Supplementary file 1 — Data S1. Supporting Information. [file BTM2-10-e70066-s001.zip › btm270066-sup-0003-Supinfo3@coating PAAm pH4.gif]

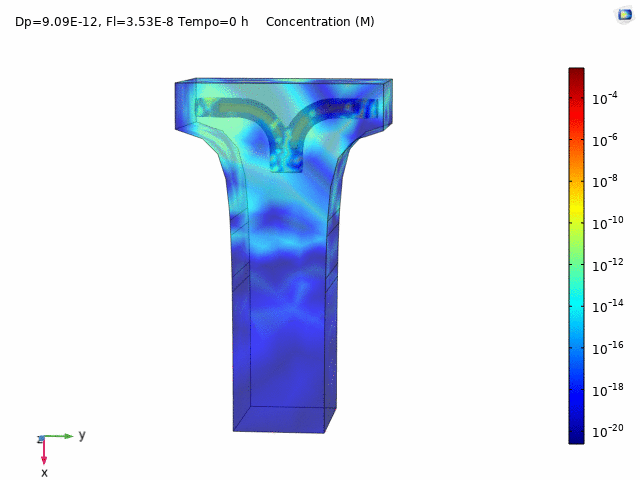

Supplement: Supplementary file 1 — Data S1. Supporting Information. [file BTM2-10-e70066-s001.zip › btm270066-sup-0004-Supinfo4@coating PAAm pH7.gif]

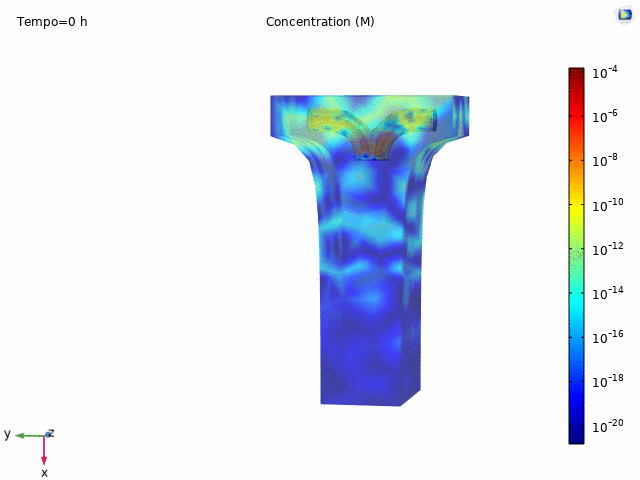

Supplement: Supplementary file 1 — Data S1. Supporting Information. [file BTM2-10-e70066-s001.zip › btm270066-sup-0005-Supinfo5@no coating pH7.gif]

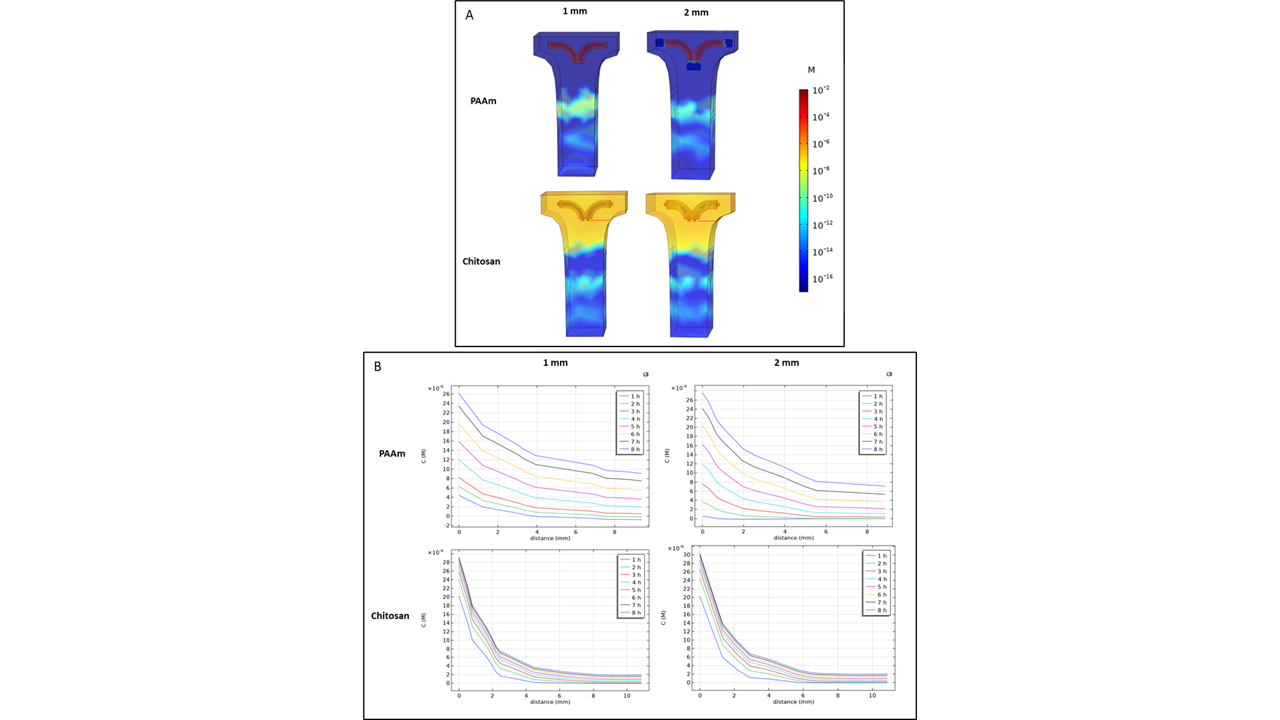

Supplement: Supplementary file 1 — Data S1. Supporting Information. [file BTM2-10-e70066-s001.zip › btm270066-sup-0006-FigureS1@Fig S1.tif]
